# Modulation of Immune Cells as a Therapy for Cutaneous Lupus Erythematosus

**DOI:** 10.3390/ijms231810706

**Published:** 2022-09-14

**Authors:** Jorge A. Soto, Felipe Melo-González, Claudia A. Riedel, Susan M. Bueno, Alexis M. Kalergis

**Affiliations:** 1 Millennium Institute of Immunology and Immunotherapy, Departamento de Genética Molecular y Microbiología, Facultad de Ciencias Biológicas, Pontificia Universidad Católica de Chile, Santiago 8331150, Chile; 2 Millennium Institute on Immunology and Immunotherapy, Departamento de Ciencias Biológicas, Facultad de Ciencias de la Vida, Universidad Andrés Bello, Santiago 8370146, Chile; 3 Departamento de Endocrinología, Facultad de Medicina, Pontificia Universidad Católica de Chile, Santiago 8330023, Chile

**Keywords:** cutaneous lupus erythematosus, systemic lupus erythematosus, immune cells, treatments

## Abstract

Cutaneous lupus erythematosus (CLE) is an autoimmune disorder like systemic lupus erythematosus (SLE). Both SLE and CLE characterize autoantibody secretion and immune cell recruitment. In particular, CLE can be divided into three more frequent types, varying in the severity of the skin lesions they present. The role of type I IFN was shown to be one of the leading causes of the development of this pathology in the skin. Different treatments have been developed and tested against these different variants of CLE to decrease the increasing levels of CLE in humans. In this article, a literature revision discussing the similarities between SLE and CLE is carried out. In addition, new advances in understanding the development of CLE and the leading treatments being evaluated in animal models and clinical trials are reviewed.

## 1. Introduction

Systemic lupus erythematosus (SLE) is an autoimmune disease that mainly affects women of childbearing age, representing around 90% of people with this pathology [1]. SLE is characterized by the production of auto-antibodies and the formation of immune complexes directed against the body itself. The associated symptoms include skin lesions with joint, renal, cardiac, and nervous system damage [2]. Renal impairment is one of the most characteristic symptoms of SLE, which is generated by the development of glomerulonephritis. It has been reported that 10% of people with SLE can develop chronic renal failure, increasing to 40% over time [3]. The prevalence of this disease is highly variable, with higher rates found in the United States compared to Africa, Europe, and Australia [4].

On the other hand, cutaneous lupus erythematosus (CLE) is an autoimmune skin disease with a wide range of clinical presentations. Multiple studies have evaluated the possibility of progressing from CLE to SLE, which has a progression rate ranging from zero to over 30% [5,6,7]. Particularly, CLE incidence is close to 4.3 per 100,000 individuals [8]. This clinical pathology is frequently identified in females, but the contribution of the hormones in the development of CLE remains unclear [9]. One of the most typical immunological factors associated with CLE induction is the development of antinuclear antibodies (ANAs) [10]. Interestingly, other types of autoantibodies have been reported, such as Ro antibodies [11] anti-smith (sm) [12], C1q [13], and HMGB1 [14]. These autoantigens can be affected by UV stimulation [15]. Moreover, the role of the keratinocyte and UVB exposure is a critical factor in the development of this disease [16].

This review consists of an update of the components associated with developing SLE and CLE, focusing on the latter. In addition, we review the most recent experimental treatments being evaluated to control the pathology caused by CLE in murine models and clinical tests.

## 2. Factors Triggering the Development of SLE

SLE development is multifactorial and is related to ethnicity, as well as genetic, environmental, hormonal, and immunological factors [17]. Among the genetic factors associated with SLE development, more than 40 polymorphisms have been found, which may affect the interferon (IFN) and IL-10 secretion pathway [18]. On the other hand, some environmental factors associated with SLE are cigarette smoking and mercury exposure, among others [19]. At the immunological level, it has been reported that dysregulation of the innate and adaptive immune system contributes to the generation of SLE [20]. The secretion of type I IFN (IFN-α) has been identified to be associated with SLE development, with elevated levels of this cytokine being found in patients with SLE [21]. IFN-α promotes a pro-inflammatory environment that activates dendritic cells (DC), natural killer (NK), macrophages, Lymphocytes T (LT), and Lymphocytes B (LB), producing a dysregulation of the immune system tolerance [22,23].

On the other hand, IL-6 is a cytokine secreted by macrophages, LT, and fibroblasts. IL-6 has been linked to the development of SLE, as it promotes the maturation of BLs to plasma cells and may affect LT activity [24]. In addition, increased IL-6 has been reported in serum samples from patients with SLE [25]. As the activation of LTs and LBs depends on some co-stimulatory signals and cytokines, changes in the control and regulation of the immune system are important to study in the context of SLE in order to identify the role of the different cells and cytokines involved in this process.

Notably, it has been described that several cells of the immune system (DCs, macrophages, and neutrophils, among others) are affected during SLE, altering their canonical functions that may impact the activation and function of LTs and LBs [26]. Alteration in the regulation of LTs may promote a polarization towards an immune profile that triggers SLE. In addition, the LBs mature towards plasma cells that secrete antibodies that attack the organism or generate immune complexes that function as signals for other immune system cells to attack our body [27,28].

## 3. SLE Can Induce Cutaneous Lesions in Patients

One of the main aspects of SLE development is the appearance of cutaneous lesions in about 80% of the patients diagnosed with this disease [29]. The apparition of the cutaneous lupus erythematosus (CLE) has been associated with a condition triggered by SLE [30] considered the first symptom of SLE in more than 25% of the population with this condition [31]. This disease is diagnosticated in 70 cases per 100,000 people [30]. CLE can be divided into three different categories lupus-dependent: acute cutaneous LE (ACLE); subacute cutaneous lupus (SCLE), and chronic cutaneous LE (CCLE) [30,32].

Some of the genetic factors that have been described to promote skin damage in SLE patients [33,34] include rs1143679 single nucleotide polymorphism (SNP) of *itgam*, which is one of the most studied genetic factors that triggers malar rash (ACLE) and discoid lupus DLE [35,36]. Moreover, an association between *ITGAM* and photosensitivity has been reported [37]. Other studies have shown that rs1801274 SNP of the *fcgr2a* gene is associated with rash malar [38], while rs11168268 SNP of the *vdr* gene is associated with cutaneous alterations in patients with SLE [39]. In particular, genetic factors involved in CLE are related to HLA class II alleles [40]. Along these lines, the variant DQA1*0102 showed an increase that promotes CCLE. Moreover, in SCLE and DLE patients, the frequency of HLA A*01, B*08, and DRB1*0301 alleles have been suggested as candidates associated with this pathology [41].

On the other hand, some inflammatory cytokines, such as TNF-α, IL-18, and type I IFN, are upregulated upon UV exposure [42,43,44]. In samples obtained from the epidermis and dermis samples of CLE patients, an increase in genes regulated by IFN were found. Moreover, type I IFNs promote the infiltration of T helper 1 (Th1) cells, which enhances pathology [45].

### 3.1. Acute Cutaneous Lupus Erythematosus (ACLE)

This condition is a dermal condition typically identified by the apparition of a butterfly rush design. It is frequently associated with UV exposition [31]. During the development of the ACLE, the occurrence of discrete or small erythematous macules, papules, and plaques visible on the face can be observed. However, other close regions such as the scalp, neck, and earlobes may be affected, showing signs of erosions, crusting, and scaling, which can confuse with other dermal conditions such as dermatomyositis [31,46,47]. ACLE patients exhibit a high association with systemic disease (up to 90%) [48], whereas up to 52% of SLE patients display ACLE [49]. In addition, a high proportion of ACLE patients (85–95%) are positive for antinuclear antibodies and display other autoantibodies against dsDNA (30–40%) and Sm, as well as local granular skin deposits of IgM, IgG, and IgA [50,51]. Therefore, this form of lupus is highly associated with systemic autoimmune disease clinical signs.

Still very little is known about the specific molecular pathways associated with ACLE. A recent study indicates that patients can be distinguished from other types of CLE as they exhibit reduced numbers of CD4^+^ tissue-resident memory T cells. In contrast, these cells are more frequently observed in subacute and chronic cutaneous lupus, promoting persistent damage [52]. Thus, ACLE pathogenesis seems to be independent of abnormal T-cell function. As mentioned above, B cells and autoantibodies seem to be associated with the pathogenesis of this type of lupus, and therapies targeting B cells appear to be effective for its treatment. The use of a monoclonal antibody against CD20, Rituximab, which leads to B cell depletion, was more effective in patients with ACLE compared to other types of cutaneous lupus and reached a 43% effectiveness (6 out of 14 patients). In contrast, different types of cutaneous lupus patients showed a poor response to the treatment, suggesting B-cell-independent mechanisms in the pathogenesis of the disease [53].

### 3.2. Subacute Cutaneous Lupus Erythematosus (SCLE)

This condition is commonly found in the V area of the neck, upper trunk, shoulders, and arms. Since the main lesion is observed, the apparition of erythematous macules or papules is the most observed. These lesions can be evolved into papulosquamous psoriasiform lesions or annular patches in almost half of the cases [31]. Association with SLE occurs in a lower frequency (20–30% of cases) than ACLE [54], suggesting different disease pathogenesis.

Genetic factors such as HLA-DR3 and HLA-DR2 have been associated with a higher production of anti-Ro antibodies, which are characteristics of this condition and are associated with UV exposure [55]. In this line, hormonal changes in estrogen have been reported to contribute to the induction of high titers of anti-Ro and anti-La antibodies and photosensitivity [56,57]. This phenomenon is explained because estrogen can enhance IgG production, especially anti-dsDNA autoantibodies, promoted by the activity of B cells and the production of IL-10 from monocytes [57].

### 3.3. Chronic Cutaneous Lupus Erythematosus (CCLE)

This pathology can be represented in different degrees of severity, which distinguishes five different categories: discoid LE (DLE), LE profundus/panniculitis (LEP), LEtumidus (LET), verrucous/hypertrophic LE, and chilblain LE (CHLE) [58]. It represents the most frequent manifestation of CLE, affecting the ears, the face, the scalp, and/or the neck. In less than 20% of the cases, an expansion of the lesions is observed, which is located mainly below the neck. Interestingly, these clinical manifestations can be increased in smokers with a complement deficiency who can spread these lesions towards the back of the hands [59]. In contrast to ACLE and SCLE, CCLE is characterized by the apparition and growth of a variable-sized erythematous plaque associated with an adherent follicular hyperkeratosis. The erythema with follicular hyperkeratosis is the first visible lesion, and then it progresses to atrophy, pigmentary changes, and scarring. These changes reported in CCLE are persistent and induce sequelae. Moreover, depending on the severity of the clinical manifestation of CCLE, patients can develop scarring alopecia on the scalp [50]. In a DLE, the cytotoxic lymphocytes can destroy stem cells of the hair follicle and cause scarring in the skin lesions [57]. Compared to other types of SLE, only 8% of CCLE patients develop systemic disease [60] whereas only 5% of DLE exhibit SLE [61]. Genetic factors can be related to susceptibility to developing DLE and SLE. It is reported that HLA-B8 is expressed in DLE patients. Interestingly, an increase in the expression of HLA-B8 has been correlated as a predictive marker of the progress from DLE to SLE [57,62].

On the other hand, transcriptional analysis of lesioned biopsies from a cohort with different types of CLE shows that patients with DLE exhibit a distinctive immunoglobulin signature and enrichment of B-cell-associated pathways in the skin compared to ACLE and SCLE patients. In addition, DLE patients exhibit more skin B cells than other types of CLE. Still, this increase does not correlate with the augmented presence of peripheral autoantibodies and SLE, suggesting that B cells play tissue-specific roles in developing DLE [63]. Another study indicates that CLE patients with and without systemic disease exhibit abnormal peripheral B-cell responses, characterized by unswitched memory and increased effector responses. However, these responses were higher in patients with systemic disease [64].

A study in patients with SCLE and DLE and healthy patients explored a possible role of microRNA (miR) during the development of the pathology. Consistent with this notion, changes in the expression levels of a wide variety of miRs expressed in lower concentrations in subjects with SCLE or DLE compared to healthy controls have been described. However, miR-21, miR-1246, and miR-150 were identified as being involved in an increase in some cell phenotypes described as CD4^+^/IL-4^+^ and CD20^+^/IL-10^+^, CD4^+^/IFN-γ cells^+^, and CD123^+^/CD196^+^/IDO^+^ [65].

The presence of miR has been identified in injured areas of volunteers with CLE. When miR-31 is overexpressed, it is associated with epidermal apoptosis mediated by positive regulation of bim, bax, p53, and casp3 genes [66,67]. In addition, miR-31 promotes an increase in NF-κB activation in keratinocytes, triggering the overexpression of pro-inflammatory cytokines such as IL-1β, IL-12, and IL-8. Additionally, miR-31 has been associated with an increase in the recruitment of innate cells (neutrophils and monocytes), which promotes the secretion of cytokines capable of favoring the recruitment of other immune cells at the site of inflammation [66,67].

On the other hand, miR-485-3p has been identified mainly in lymphocytes and fibroblasts recruited in the injured areas. The role of miR-485-3p promotes the activation of CD4^+^ and CD8^+^ lymphocytes. In addition, miR-485-3p has been related to a protective response against the development of fibrosis through peroxisome PPARGC1A [66,67]. Therefore, miR has been mainly associated with pro-inflammatory effects in CLE pathogenesis [66,67].

## 4. Immunological Involvement in the Development of CLE

**Figure 1:**
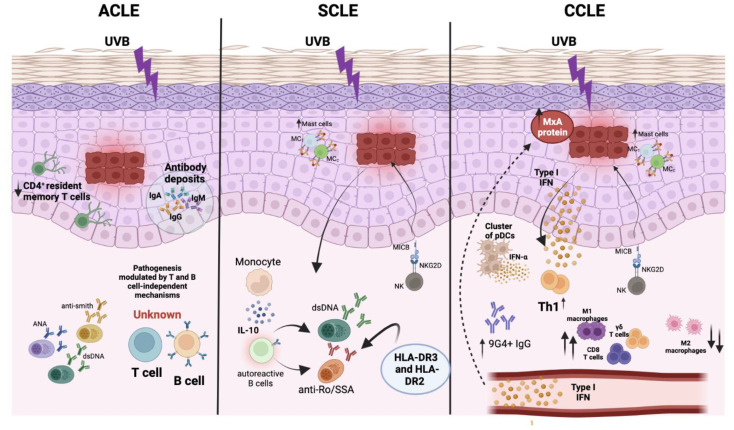
The immune cells are involved in the induction of CLE. The figure shows the main immunological mechanisms associated with the induction of ACLE (left panel), SCLE (middle panel), and CCLE (right panel) in the presence of a stimulus with UVB light. The arrows pointing up symbolize an increase, while the arrow pointing down represents a decrease.

One of the most typical immunological factors associated with CLE induction is the development of antinuclear antibodies (ANAs). However, many CLE patients also develop anti-Ro/SSA (anti-Sjogren’s syndrome-related antigen A) [10]. The Ro antibodies are associated with two different ribonucleoproteins isotypes, TRIM21 (52 KDa) and TROVE2 (60 KDa) [68], where the latter is the most frequently found in CLE patients [11]. However, almost three other antigens can be recognized by the immune system in CLE patients, triggering the induction of autoantibodies. These molecules are known as smith (sm) antigen [12], C1q [13], and HMGB1 [14]. UV stimulation can affect these autoantigens, promoting the translocation towards the keratinocyte surface, facilitating the recognition of these antigens by the immune system [15]. Although these secreted autoantibodies can induce CLE in patients, their presence can modulate the different CLE in the patients. In this line, CCLE is characterized as not presenting extractable nuclear antigens (ENA), anti-dsDNA, or anti-Ro/SSA. At the same time, SCLE promotes the induction of anti-smith, anti-Ro/SSA, anti-RNP, and ENA autoantibodies. Finally, ACLE patients showed an increase in the anti-dsDNA and ANA autoantibodies as in SLE patients, where females are frequently affected (Figure 1) [69].

On the other hand, the immune cells are essential to induce damage and collaborate with CLE development. Once keratinocytes are affected by UVB radiation or drugs, this promotes the activation of immune cells, which triggers a pathogenesis effect in the skin. Moreover, keratinocytes can modulate the activation of absent in melanoma 2 (AIM2) inflammasome through MDA5, RIG-I, c-GAS, and STING, but not TLR-independent nucleic acid ligands [48].

Langerhans cells (LC) are important antigen-presenting cells that reside in the epidermis and display dendritic-cell-like functions. In a murine model, during the stimulation of keratinocytes with UVB radiation, LC upregulated ADAM17, which increases the availability of the active form of epidermal growth factor receptor (EGFR) ligands to avoid damage to the keratinocytes (Figure 1) [70]. Interestingly, in an SLE murine model, the activation of the EGFR pathway was affected by an impaired role of the LC, triggering an increase in the cutaneous lesion of these mice [71]. These results are supported by a recent study of CLE patients, which showed that microarray assays in biopsies from CLE patients decreased EGFR signaling pathways [72]. Another DC subtype involved in CLE is plasmacytoid DCs (pDCs), which are not typically found in the skin [73]. pDCs are essential to initiate the pro-inflammatory pathogenesis induced by skin infections and autoimmune diseases [74]. These cells are mainly found in DLE patients, promoting upregulation of IFN-α [75]. It has been reported that pDCs are more so recruited in the presence of UVB, considered an essential source of type I IFN to develop skin lesions [76,77]. Additionally, the interaction between the HSP70 receptor Lox-1 presented in pDCs with HSP70 expressed in keratinocytes promotes an increase in the uptake of exogenous DNA, which promotes an increase in the type I IFN secretion by pDCs [78] (Figure 1). This effect is observed in CLE and vitiligo patients [78]. On the other hand, CD16+ DCs have been reported in SLE, triggering a robust interferon response in the skin, which could be related to CLE pathogenesis [79].

Interestingly, it has been described that IFN-κ is fundamental in the development of CLE since it can enhance the type I IFN response, which is critical for promoting the pathology [80]. In addition, these molecules show an enhanced response to photosensitivity in keratinocytes against exposure to UV light [80]. As expected, the increased response to type I IFN induced against skin lesions promotes a CD4^+^ polarization profile towards a Th1 response. The importance of the IFN type I response and the polarization profile was evaluated in biopsies from patients with CLE, identifying an increase in the IP10/CXCL10 and CXCR3 characteristic of the Th1 response and the MxA protein, which is induced by type I IFN response [45].

The use of knockout mice for immune inhibitory receptor VISTA or programmed death-1 homolog (PD-1H KO) on a BALB/c background showed a spontaneous development of cutaneous and systemic autoimmune lupus. Notably, in CLE, lesions of PD-1H KO mice were characterized by a cluster of pDCs similar to human DLE. Moreover, in this model, it was possible to define that neutrophils are critical for early immune infiltrating cells in CLE, suggesting that PD-1H constitutes an essential element involved in the pathogenesis and progression of SLE and CLE [81].

Another immune cell involved in CLE development is the T cell, and its different phenotypes have been evaluated in CLE patients. In SCLE patients, a significant decrease in CD4 T cells and CD4/CD8 ratio (Figure 1) was found in comparison to DLE and erythematosus lupus tumidus (LET). Further, a low number of FOXP3^+^ and CD39^+^ T helper cells were found in SCLE and LET concerning DLE patients, suggesting that Tregs cells are mainly affected in the more photosensitive CLE phenotypes [82].

On the other hand, the role of different cytokines was evaluated in serum from SLE and CLE patients. This study detected an increase in the IL-6 cytokine in DLE and SLE patients compared to the control group [83]. This effect was similar for IL-23, which increased in DLE and SLE patients compared to the control group. Interestingly, the IL-17/IL-10 ratio was also evaluated, showing that DLE and SLE presented an increase in this ratio compared to the control group. These results suggest that CLE and SLE induce a Th17 profile over the Treg profile (Figure 1). In this line, the development of therapies against these cytokines could be a target to decrease the pathology associated with this autoimmune disorder [83].

Another population of T cells involved in CLE is the CD4^+^ tissue-resident memory T (CD4^+^ Trm). These cells were evaluated in lesions from ACLE, SCLE, and DLE patients [52]. In this study, it was identified that CD4^+^ Trm cells were raised in the lesion of both SCLE and DLE concerning ACLE. Furthermore, the increase in CD4^+^ Trm cells was correlated with persistence in the lesions of SCLE and DLE. Further, the authors found that AIM2 expression in CD4^+^ Trm cells can discriminate between the affected groups of SCLE and DLE compared to ACLE patients [52].

Moreover, it has been reported that the NKG2D receptor is strongly expressed in DLE and SCLE [84]. The detection of the NKG2D receptor, which is commonly found on NK cells and some T-cell populations [85,86] was related to an increase in the lymphocytic infiltrate in DLE and SCLE [84]. These cells are recruited in skin lesions caused by stressed keratinocytes, which overexpressed the MICB ligand, which is recognized by the NKG2D receptor promoting skin damage [84].

The mast cells (MCs) are another type of immune cell that contribute to different lupus erythematosus diagnostics associated with metalloproteinase production and contribute to immune cell migration and tissue damage [87]. In this line, MCs are divided into three different subsets: the chymase and tryptase (MC_TC_); tryptase-positive (MC_T_), which only contain tryptase; and the only chymase-positive mast cells (MC_C_) [88,89,90]. In particular, the MC_TC_ are predominant in the skin [91]. A study comparing MCs’ infiltration into skin biopsies from SLE, DLE, and SCLE found the highest infiltration of both chymase-positive and tryptase-positive mast cells in SCLE and DLE patients. In contrast, a minor infiltration was found in SLE patients [92].

B cells were evaluated using a humanized murine model. Here, the age-associated B cell (ABC, T-bet+ CD11b+) group in response to IL-21 and TLR7/9 signals promotes the recruitment of autoreactive B cells, which produces IgG2a, IgG2b, and IgG3 antibodies, which exacerbate inflammation and thus drive lupus-like autoimmunity [93] (Figure 1). Another study showed that CCLE+/SLE− patients share B-cell abnormalities with SLE patients, including increased effector B cells and decreased unswitched memory. Additionally, SLE and CCLE+/SLE− patients presented elevated 9G4+ IgG autoantibodies accompanied by lower levels of anti-nucleic acid and anti-RBP antibodies [64].

To understand the role of B cells in CLE patients, a study evaluated the differences in the expression profiles of these cells in DLE and SCLE patients using an autoimmune profiling panel (NanoString) [94]. DLE patients showed an upregulated expression of CD19, CD20, and CD79a. Moreover, an increase in protein production was found in immunoglobulins, B-cell-activating factor (BAFF) receptors, and members of the Fc-receptor-like (FCRL) family [94].

Bioinformatic analyses from DLE and healthy control groups were evaluated to understand the role of different immune system components in DLE patients [95]. Immune filtration analyses using the CIBERSORT database showed that DLE samples had a significant increase in the number of CD8^+^ T cells, memory-activated CD4 T cells, γδ T cells, and M1 macrophages, but reduced numbers of regulatory T cells and M2 macrophages [95]. Moreover, lower resting DCs, mast cells, and activated mast cells were found compared to samples from healthy patients [95].

## 5. Current Treatments against Cutaneous Lupus

The first recommended treatment for localized cutaneous lupus lesions is topical corticosteroids for short-term and prolonged use in scalp lesions. A randomized controlled trial conducted in patients with DLE treated with a potent corticosteroid cream composed of fluoxinide 0.05% was efficacious in 27% of cases compared to a 10% efficacy using a low-potency steroid, 1% hydrocortisone [96]. In addition, a pilot study in DLE patients showed a 73% efficacy using betamethasone 17-valerate 0.1% (73%) for treating facial lesions [97]. However, this latter treatment was not significantly different from the other alternative treatment with the calcineurin inhibitor pimecrolimus 1%, exhibiting an 86% efficacy in DLE patients. Furthermore, the topical use of tacrolimus 0.1% and 0.03 %, other calcineurin inhibitors, has also shown to be highly effective in cutaneous lupus lesions [98] and facial DLE lesions [99]. Thus, steroids and calcineurin inhibitors can be used to treat localized cutaneous lupus lesions, especially in DLE patients.

On the other hand, CLE patients are currently treated with antimalarials such as hydroxychloroquine (HCQ) and quinacrine as the first line of treatment and have been used for decades in more than 70 countries. For example, a phase 3 trial in Japan reported that using HCQ in active CLE patients was safe up to 55 weeks of treatment and improved the clinical signs of disease at week 16 [100]. However, these drugs presented several adverse effects and the potential risk of retinopathy [101], as well as with the presence of a particular group of T cells expressing CD69+CCR7+ and STAT3 [102].

Another study compared the use of HCQ and quinacrine (QC) [103], observing that HCQ responders increased CD4^+^ T cells compared to QC. Moreover, HCQ presented lower central memory T cells than control groups, while QC responders showed higher T regs compared control group. Furthermore, in the QC group, an increase in the phosphorylated (p) STING and IFNκ was found with respect to HCQ, suggesting that CLE patients can present differential immune compositions [103].

Other drugs tested in different CLE subtypes are immunomodulatory imide drugs (IMID), including thalidomide and lenalidomide, which can modulate innate and adaptive immune responses reported in CLE, preventing in particular inflammatory cytokine production and modulating Treg responses [104]. A meta-analysis of 21 studies among 548 patients showed a 90% response in different CLE subtypes, especially in patients with DLE and SCLE, but the pooled rate of thalidomide withdrawal was 24% due to adverse effects including neuropathy and thromboembolic events. These results suggest that the use of thalidomide should be avoided due to a neurotoxicity effect in patients with severely refractory CLE or who are at high risk for severe scarring [105]. A similar IMID, lenalidomide, has shown efficacy in cases of severe refractory CLE with low frequency of neuropathy, which could represent an alternative to the use of thalidomide [106].

Several biological agents have been tested for treating CLE patients but with inconclusive results. Belimumab, a monoclonal antibody against BAFF, has been tested in a phase 3 trial for SLE patients who showed improvement in their mucocutaneous lesions [107]. In line with this, a small study with 16 CLE patients who were refractory to other treatments showed some response to the therapy with belimumab, especially those with mild and persistent lesions [108]. Although treatment with the depleting B-cell antibody rituximab has shown efficacy in treating SLE cases, only patients exhibiting acute and active cutaneous disease benefit from the treatment, whereas those with SCLE and CCLE have shown poor improvement in their skin lesions [109]. In addition, SLE patients with renal involvement are more likely to respond to rituximab treatment, whereas those with lower disease activity scores are less likely to improve [110].

Moreover, treatment with ustekinumab, which inhibits the pro-inflammatory cytokines IL-12 and IL-23, was shown to be effective in one patient with SCLE [111] whereas a phase 2 study showed that this treatment was safe in patients with active SLE and improved mucocutaneous disease [112] remains to be elucidated whether this treatment is more effective against certain types of cutaneous lupus. Finally, the use of BDCA2 antibody (BIIB059) was evaluated in clinical trials showing a decrease in the type I IFN by pDCs reducing the immune infiltrates in skin lesions [113].

**Figure 2:**
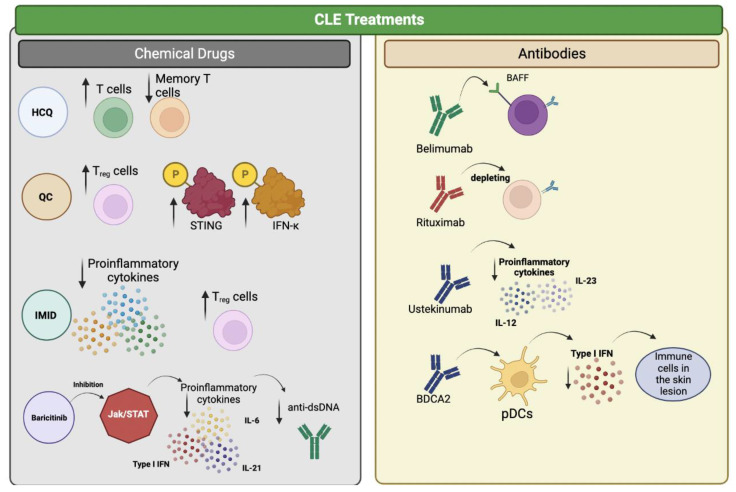
Treatments against CLE. The figure shows different treatments evaluated in humans and their interaction with the immune system. Treatments based on chemical compounds are shown in the left panel. Monoclonal antibodies evaluated as therapy against CLE are shown in the right panel. The arrows pointing up symbolize an increase, while the arrow pointing down represents a decrease.

Other biological molecules used for the treatment of SLE are inhibitors of the JAK/STAT pathway, which can prevent the secretion of pro-inflammatory cytokines, such as IL-6, IL-21, and type I IFN [114]. A molecule described as a selective and reversible inhibitor of JAK1 and JAK2 pathway, baricitinib, was tested in a phase 2 trial in SLE patients, but no difference in disease score was reported [115]. However, another clinical trial showed that decreases the presence of anti-dsDNA in SLE patients [116], whereas case reports have reported that is effective for the treatment of skin lesions in cases of SLE and SCLE patients [117,118,119]. Therefore, the use of baricitinib may be a promising alternative for the treatment of cutaneous lesions in SCLE patients but phase 2 and 3 clinical trials are required to further investigate its efficacy (Figure 2).

## 6. Conclusions

CLE is a disorder involving many factors similar to those described for SLE, which reinforces the idea that these two autoimmune pathologies may be a predisposing factor for the appearance of the other pathology. In addition, different immunological components such as cells and cytokines have been identified in both the different types of CLE and SLE; it is necessary to identify factors specific to each pathology. In this sense, directing the new studies to pathology-specific analyses that allow discriminating specific roles of cells during the progression of the disease is key to developing better treatments to reduce severe cases of these diseases. Finally, the new therapies under evaluation suggest promising effects in reducing CLE symptoms. In addition, these treatments could positively impact reducing the inflammatory processes associated with different skin diseases, triggered not only by CLE but also by SLE, psoriasis, or other pathologies of autoimmune origin.
